# Modeling and Optimization of Safflower Seed (*Carthamus tinctorius* L.) Alkaline Pretreatment Parameters to Increase Oil Yield

**DOI:** 10.3390/foods15152730

**Published:** 2026-08-03

**Authors:** Alexandr Borovskiy, Sayagul Tazhina, Gaukhar Akshorayeva, Bakhyt Shaimenova, Amirsana Kiykbay, Linara Murat, Seitkamal Yermekbayev, Zhuldyz Satayeva, Gulnazym Ospankulova

**Affiliations:** 1 Department of Food Technology and Processing Products, Technical Faculty, Saken Seifullin Kazakh Agrotechnical University, Zhenis Avenue, 62, Astana 010000, Kazakhstan; a.yu.borovskiy@gmail.com (A.B.); sayagultazhina@gmail.com (S.T.); gaukharmadok@gmail.com (G.A.); bshaymenova@mail.ru (B.S.); sanabayq@gmail.com (A.K.); linaraazamatkyzy@mail.ru (L.M.); seitkamal.yermekbayev1956@gmail.com (S.Y.); julduz.kaynar@mail.ru (Z.S.); 2 Aron R&D Co., LLP, Akmeshit Street, 5, Astana 010000, Kazakhstan

**Keywords:** safflower seed, alkaline pretreatment, response surface methodology (RSM), oil yield

## Abstract

Safflower seed is a valuable source of vegetable oil. Despite current advances in processing technology, the oil extraction efficiency during cold pressing remains relatively low, which is attributable to the seed morphology and the dense seed hull that acts as a barrier. Traditional oil extraction methods are relatively inefficient; therefore, considerable attention is focused on seed pretreatment prior to oil pressing, as this stage plays a critical role in the overall oil production process. The possibility of increasing oil yield by treating safflower seeds prior to pressing was studied using response surface methodology (RSM). Three factors were investigated: NaOH solution concentration from 1% to 2%, treatment temperature from 40 °C to 60 °C, and treatment time interval from 40 min to 60 min. A Box–Behnken design was applied to evaluate the effect of the three independent variables on oil yield. Following a series of experiments, the RSM tool was applied, which identified the optimal pretreatment parameters: NaOH concentration—1.64%, temperature—53.54 °C, and duration—51.11 min. The predicted oil yield was 20.5%, while the experimentally determined oil yield under optimal conditions reached 21.47%. The application of response surface methodology increased the oil extraction efficiency by 37.52%.

## 1. Introduction

Vegetable oils are an important component of the human diet, serving not only as an energy source but also providing essential fatty acids, tocopherols, and other biologically active compounds [1].

Safflower (*Carthamus tinctorius* L.) is regarded as a valuable source of vegetable oil, characterized by high adaptability to arid climatic conditions and the ability to utilize moisture from deep soil layers, which advantageously distinguishes it from many conventional agricultural crops [2].

Safflower seeds are characterized by a valuable chemical composition; they contain lipids, proteins, tocopherols, and phenolic compounds [3]. The oil content of safflower seeds, depending on cultivar characteristics and growing conditions, ranges from 20 to 48%; at the same time, a significant proportion of seed mass is accounted for by the hull, which can constitute up to 50–60% of the seed, substantially limiting access to the lipid fraction [4]. The particularly high nutritional and biological value of safflower oil is determined by its elevated content of unsaturated fatty acids, primarily linoleic acid, which can reach 70–80% [5,6].

Scientific investigations also highlight its antioxidant, hypolipidemic, and anti-inflammatory properties, which broaden the application potential of this oil in the food, pharmaceutical, and related industries [7,8,9,10].

Despite the high technological and biological potential of safflower as an oilseed crop, the efficiency of oil extraction from its seeds remains insufficient. One of the primary factors reducing oil yield is the specific morphological structure of the seeds. The dense hull, formed predominantly by lignified tissues with a high number of wax and cutin compounds, creates a pronounced barrier that impedes mass transfer processes and restricts the access of extracting or mechanical forces to intracellular lipid structures. As a result, a portion of the oil is retained in the cake after processing, reducing the overall yield of the target product and the economic efficiency of the process [11,12,13,14].

In this regard, the use of conventional oil extraction methods alone does not always ensure a high yield of the target product: mechanical pressing frequently fails to achieve complete disruption of cellular structures, with the result that a portion of the oil remains bound within the plant matrix and passes into the cake. Organic solvent extraction, despite its higher efficiency, also has significant limitations associated with the use of potentially toxic reagents, the necessity of their subsequent removal, and increased energy consumption [15]. In this context, pretreatment of safflower seeds is considered a necessary raw material preparation step that creates more favorable conditions for subsequent oil extraction.

Existing methods for the pretreatment of plant raw materials are classified as physical, biological, and chemical. Physical methods (grinding, thermal and microwave treatment) provide partial disruption of cellular structures but are characterized by high energy consumption and limited efficiency in the removal of antinutrients [16]. Biological methods (enzymatic and microbiological treatment) are highly selective but require considerable time and are difficult to scale up [17].

The most promising approach is chemical pretreatment, which includes the use of acids, alkalis, oxidizing agents, and organic solvents. Alkaline treatment of lignocellulosic plant raw materials possesses a number of significant advantages over acid treatment [18]. Unlike acid treatment, which primarily targets hemicellulose hydrolysis and may partially degrade the cellulose matrix, alkaline treatment predominantly interacts with lignin, resulting in delignification of the lignocellulosic raw material [18,19]. It is also known that alkaline treatment induces swelling of cell walls and promotes cleavage of bonds between lignin and carbohydrates [20], thereby enhancing mass transfer and increasing oil yield. Furthermore, alkaline treatment promotes a reduction in antinutrient content, such as phytic acid [21], and decreases bitterness, thus improving the feed value of safflower meal. Alkaline treatment has been successfully applied to various oilseed crops, including sesame and hazelnut, where optimization of treatment parameters (alkali concentration, temperature, and time) allows for a substantial improvement in raw material quality [22,23,24,25].

It should be noted that the efficacy of alkali application during seed preparation for pressing is determined by a combination of technological parameters, including solution concentration, temperature, and treatment duration. Excessive values of these parameters can lead to lipid saponification and deterioration of oil quality. Therefore, optimization of the pretreatment process conditions is a key objective. In contemporary research, response surface methodology (RSM) is widely employed for this purpose, enabling modeling of factor effects and identification of optimal conditions with a minimum number of experiments [26].

Despite the existence of research in the field of plant raw material pretreatment, the application of alkaline treatment to safflower seeds remains poorly studied. In particular, systematic investigations aimed at comprehensive process parameter optimization using mathematical modeling methods are lacking, and the effects of alkaline pretreatment on the structural changes in the hull and oil yield are insufficiently characterized.

Based on the foregoing, the objective of this study was formulated as follows: mathematical modeling of the safflower seed preparation process for oil extraction using sodium hydroxide by means of response surface methodology and optimization of the process under investigation.

## 2. Materials and Methods

### 2.1. Materials, Chemicals, and Reagents

Safflower seeds (*Carthamus tinctorius* L.) of the cultivar “Möldır-2008”, provided by Krasnovodopadskoye Agricultural Experimental Station LLP, were used as the study object. The oil content of the seeds under investigation was 25.61 ± 0.6%. Prior to treatment, seeds were stored under dry conditions at room temperature.

All chemicals and reagents were of analytical grade and were produced by Sigma-Aldrich (St. Louis, MO, USA).

### 2.2. Raw Material Preparation and Alkaline Pretreatment

Safflower seeds were preliminarily cleaned of mechanical impurities (dust, plant debris, damaged seeds) manually and using a laboratory sieve.

For physicochemical analyses, seeds were additionally ground using a laboratory mill (LZM-1, NV-LAB, Moscow, Russia) and sieved through an 18-mesh sieve to ensure sample homogeneity and improve the accuracy of analytical measurements. Prepared samples were stored in sealed containers at 4 ± 1 °C until the experiments were conducted, minimizing exposure to light and oxygen to prevent oxidative processes.

Alkaline treatment was carried out at a fixed liquid-to-solid ratio of 2 mL/g. Seeds were placed in conical flasks, covered with alkali solutions of the specified concentration, and incubated in a thermostat at the corresponding temperature and time. Upon completion of the process, the raw material was thoroughly rinsed with warm water until the alkali was completely removed, as monitored by pH, and washed with a dilute citric acid solution (0.1%, 40 °C), which neutralized traces of alkali and soap compounds. The seeds were then additionally rinsed with clean water, drained through a sieve, and dried in a thin layer in a drying oven at 40 °C to an air-dry state, ensuring a moisture content of 6–8%.

### 2.3. Experimental Design

#### 2.3.1. Selection of Factor Levels

To investigate the effect of technological parameters of alkaline treatment on safflower seed oil yield, single-factor experiments were conducted using alkali solutions of varying concentrations while varying treatment temperature and duration.

The following factors were varied in the experiments:

NaOH concentration of the alkali solution—0.5; 1.0; 1.5; 2.0 and 2.5%;

Treatment temperature—20, 30, 40, 50, 60, 70, 80, 90 and 100 °C;

Treatment duration—10, 20, 30, 40, 50, 60, 70 and 80 min.

#### 2.3.2. Optimization of the Pretreatment Process

To optimize the alkaline pretreatment of safflower seeds for increasing oil yield (Y, %), response surface methodology (RSM) with a Box–Behnken design was applied, employing three independent variables: alkali concentration (X1, %), treatment temperature (X2, °C), and treatment duration (X3, min). The choice of this design was due to its efficiency for constructing quadratic models with a relatively small number of experiments and to the absence of extreme corner points.

The full design included 15 experimental points, including 3 central replicates for estimating the pure error and lack-of-fit. The run order was randomized.

The experimental data were fitted with a quadratic polynomial model, Formula (1):

(1) $$Y=\beta_{0}+\sum_{i=1}^{3}\beta_{i}X_{i}+\sum_{i=1}^{3}\beta_{ii}X_{i}^{2}+\sum_{i=1}^{3}\times \sum_{j=i+1}^{3}\beta_{ij}X_{i}X_{j}+\varepsilon$$

where *Y* is the response (oil yield, %); *β*_0_ is the constant; *β*_i_, *β*_ii_, *β*_ij_ are the coefficients of the linear, quadratic and interaction terms; and ε is the error.

#### 2.3.3. Oil Extraction

Cold pressing of safflower seeds was carried out using a laboratory cold press (USOP 10 (Universal screw oil press), capacity 12 kg/h, single head, 4 kW, Agrotehservis-12, Kostanay, Kazakhstan) according to the method of [27] with modifications. The following constant pressing parameters were selected: screw rotation speed of 40 rpm and maximum outlet temperature of 40 °C. After pressing, the oil was filtered through Whatman No. 1 filter paper. The purified oil was weighed on an analytical balance, after which the yield was calculated.

Oil yield was calculated as the ratio of the mass of extracted oil to the mass of the initial raw material, according to the following equation (Equation (2)) [28]:

(2) $$Y=\frac{m1}{m2}\;\times \;100,\%$$

where m_1_—mass of extracted oil, g; m_2_—mass of initial sample, g.

Oil extraction efficiency was calculated as the ratio of oil yield to oil content according to Equation (3) as described in [29]:

(3) $$O=\frac{m1}{m2}\;\times \;100,\%$$

where m_1_—oil yield, %; m_2_—oil content, %.

### 2.4. Determination of the Main Physicochemical and Structural Characteristics of Safflower Seeds and Oil Before and After Pretreatment

To assess the effect of alkaline pretreatment on safflower seed quality, the main physicochemical parameters of the control sample (CS) and the optimized sample (OS)—seeds treated with an alkali solution under the established optimal technological conditions—were investigated.

#### 2.4.1. Determination of Residual Alkalinity in the Aqueous Extract of Seeds After Alkaline Treatment

Residual alkalinity after alkaline treatment was determined according to the method described by [30]. Safflower seed samples were ground and extracted for 1 h at 20 ± 2 °C with 1 N NaCl solution. Consequently, the extracts were filtered and titrated with 0.1 N HCl using phenolphthalein as the endpoint indicator.

#### 2.4.2. Determination of Hull Content

Hull (shell) content was determined as described in [31] by a gravimetric method with modifications. A 10 g sample was taken from the representative sample. The hull was separated from the kernel manually using tweezers, ensuring complete hull removal without kernel losses. Preliminary light mechanical scoring of the hull was permitted to facilitate separation. The separated hulls were collected separately and weighed on an analytical balance to the nearest 0.001 g. The hull-to-seed ratio was calculated according to Equation (4):

(4) $$Htotal=\frac{m1}{m2}\;\times \;100$$

where m_1_—the hull mass, g; m_2_—the mass of the initial seed sample, g.

#### 2.4.3. Determination of Oil Color

The color characteristics of the samples were determined using a benchtop YS6010 spectrophotometer (3nh Technology Co., Guangzhou, China) in accordance with a previously described method [32], with modifications.

#### 2.4.4. Determination of the Acid Value of the Oil

The acid value (Av) of safflower seeds was determined in accordance with the method described by Sarkar et al. [33], with modifications. In total, 1.0 g of oil was placed in a flask and completely dissolved in 25 mL of 95% ethanol. A few drops of 1% alcoholic phenolphthalein solution were added to the resulting solution, and it was titrated with 0.1 mol/dm^3^ KOH solution until a stable pale-pink color appeared that persisted for at least 10 s. The volume of potassium hydroxide solution (V, KOH) consumed for the titration was recorded. The acid value was expressed in milligrams of KOH and calculated by Equation (5):

(5) $$Av=\frac{V\;\times \;c\;\times \;56.1}{m},$$

where *Av* is the acid value (mg KOH/g); *V* is the volume of KOH solution consumed for the titration (cm^3^); c is the molar concentration of the KOH solution (mol/dm^3^); 56.1 is the molar mass of KOH; m is the mass of the sample portion (g).

#### 2.4.5. Determination of the Peroxide Value of the Oil

The peroxide value (Pv) of the oil was determined by the method of Dehghan et al. [34], with minor modifications. A 2.0 g portion of oil was mixed with 50 mL of chloroform and thoroughly stirred; the resulting mixture was filtered and a 20 mL aliquot was taken, to which 30 mL of acetic acid was added. In total, 1 mL of saturated potassium iodide solution was added to the mixture, after which it was kept in the dark for 30 min. Then 2 mL of 1% starch solution and 50 mL of distilled water were added, and the mixture was titrated with 0.01 N sodium thiosulfate solution until decolorization. The peroxide value was calculated by Equation (6):

(6) $$Pv=\frac{V\;\times \;N\;\times \;1000}{m}$$

where *V* is the volume of sodium thiosulfate solution consumed for titrating the sample, and m is the mass of the oil.

#### 2.4.6. Determination of the Saponification Value of the Oil

The saponification value of safflower oil was determined in accordance with the method described by Nabede et al. [35]. In total, 2 g of oil was placed in a flask and mixed with 25 mL of 0.5 N KOH solution. After boiling for 1 h, the flask was cooled under running water. Then 2–3 drops of phenolphthalein were added and titration was carried out with 0.5 N HCl solution until the disappearance of the pink color and restoration of the initial color of the mixture. In parallel, a blank experiment was carried out following the same procedure, using 2 mL of distilled water instead of the oil sample. The saponification value was calculated by Formula (7):

(7) $$SV=\frac{(V_{0}\;-\;V)\;\times \;N\;\times \;56.11}{P}$$

where *SV* is the saponification value of the oil (mg KOH/g); *V*_0_ is the volume of HCl (mL) consumed in the blank experiment; *V* is the volume of HCl (mL) consumed in titrating the sample; *P* is the mass of the sample (g).

#### 2.4.7. Determination of the Total Content of Phenolic Compounds

The total content of phenolic compounds in safflower oil was measured by the method described in [36], with minor modifications. A 1.5 g portion of oil was dissolved in 1.5 mL of hexane and extracted with 1.5 mL of aqueous methanol solution (ethanol–water in a ratio of 80:20) and centrifuged at 3000 rpm for 10 min three times. After centrifugation, the supernatant was carefully collected with a pipette, and a 500 µL volume of the obtained extract was transferred into a 10 mL volumetric flask. In total, 0.5 mL of Folin–Ciocalteu reagent and 2 mL of saturated sodium carbonate solution (4%) were also added to the calibration flask, shaken for 3 min, and then stored in the dark for 1 h. Measurement was performed at 760 nm using a Shimadzu UV1900i spectrophotometer (Shimadzu, Kyoto, Japan). Gallic acid was used as the reference standard. Each sample was analyzed in triplicate. Gallic acid calibration curves were prepared with a concentration range of 1–10 mg/mL, yielding the equation y = 0.1016x − 0.0586 (R^2^ = 0.994). The results were expressed as mg of gallic acid equivalents/kg (mg GAE/kg).

#### 2.4.8. Determination of the Antioxidant Activity of the Oil

The antioxidant activity of safflower oil was evaluated using two methods: determination of the scavenging capacity of the 2,2-diphenyl-1-picrylhydrazyl (DPPH) free radical and of the 2,2′-azino-bis(3-ethylbenzothiazoline-6-sulfonic acid) (ABTS) radical according to the method of Ncab et al. [37], w ith minor modifications. To prepare each sample, 5 g of safflower oil was mixed with 5 mL of methanol, after which it was centrifuged at 4500 rpm for 5 min, and the supernatant—the methanol extract—was collected. The extraction was repeated two more times. The obtained supernatants were combined and stored at a temperature of 4 °C until the antioxidant activity analysis.

##### Determination of DPPH Radical Scavenging Capacity

A 2 mL aliquot of the supernatant, prepared as described above, was mixed with 2 mL of a DPPH solution in methanol with a concentration of 0.1 mmol/L. The mixture was kept in the dark at room temperature for 30 min. The decrease in optical density was measured at a wavelength of 517 nm using a Shimadzu UV1900i spectrophotometer (Shimadzu, Kyoto, Japan). Trolox was used as the standard substance, and the results were expressed in µmol of Trolox equivalent per 100 g of oil (µmol TE/100 g). Measurements of each sample were carried out in triplicate.

##### Determination of ABTS Radical Scavenging Capacity

In the course of the study, an analysis using the ABTS radical was carried out. The ABTS free radical solution was prepared as follows. An ABTS solution with a concentration of 7.0 mM was mixed with a potassium persulfate (K_2_S_2_O_8_) solution with a concentration of 2.4 mM, after which the resulting mixture was kept in the dark for 12–16 h. Then the solution was diluted with methanol in a ratio of 1:1 (*v*/*v*) at pH 7.4 until a stable optical density of 0.7 at a wavelength of 734 nm was reached.

Next, 100 µL of the methanol extract—the supernatant of each oil sample—was mixed with 1.0 mL of ABTS free radical solution and 1.0 mL of methanol in the dark. After 6 min, the optical density was measured using a Shimadzu UV1900i spectrophotometer (Shimadzu, Kyoto, Japan). Trolox was used as the standard substance, and the results were expressed in µmol of Trolox equivalent per 100 g of oil (µmol TE/100 g). Each methanol extract was analyzed in triplicate.

#### 2.4.9. Determination of the Fatty Acid Composition of the Oil

##### Preparation of Fatty Acid Methyl Esters (FAME)

Fatty acid composition was determined after conversion of lipids into fatty acid methyl esters (FAME) according to the method of Göncüoğlu et al. [38], with minor modifications. Briefly, 0.5 g of sample was weighed into glass centrifuge tubes and extracted with hexane three times (5 mL each). After each extraction, samples were centrifuged at 5340× *g* (equivalent to 7500 rpm) for 3 min, and the supernatants were pooled.

An aliquot of 10 mL of the combined hexane extract was transferred to a clean tube, and 100 µL of 2 N KOH in methanol was added to perform transesterification. The mixture was vortexed for 1 min and centrifuged at 5340× *g* for 3 min. The upper hexane phase containing FAME was carefully collected and filtered through a 0.45 µm PTFE filter into GC vials.

FAME analysis was performed using a gas chromatograph equipped with a flame ionization detector (GC-FID; Agilent 6890N, Agilent Technologies, Waldbronn, Germany). Separation was carried out on a DB-23 capillary column (60 m × 0.25 mm, film thickness 0.25 µm; Agilent J&W, Santa Clara, CA, USA).

Injector temperature was 250 °C and detector temperature was 260 °C. Samples (1 µL) were injected in split mode (1:50). Helium was used as the carrier gas at a constant flow rate of 1.0 mL/min. The oven temperature program was set to 160 °C for 5 min, followed by an increase to 220 °C at a rate of 5 °C/min, and then held at 220 °C for 8 min.

Commercial FAME standards were used for peak identification. Fatty acids were identified by comparing retention times with authentic standards. Quantification was performed using peak area normalization, and results were expressed as relative percentages of the total detected fatty acids. Fatty acids below 1% were not considered.

### 2.5. Statistical Analysis

Statistical analysis was performed in Minitab version 22 (Minitab Inc., State College, PA, USA). For each factor, one-way analysis of variance (One-way ANOVA) was applied. The normality of the data distribution was assessed using probability plots. The equality of variances was checked using Bartlett’s test. Since the ANOVA assumptions were met, parametric analysis of variance followed by a post hoc Tukey test at a significance level of α = 0.05 was used for multiple comparisons of the means. The results are presented as the mean value ± standard deviation *(n* = 3). The significance of the model and its terms was assessed by ANOVA (F-test, *p*-value < 0.05). The adequacy of the model was checked by the coefficients of determination (R^2^, R^2^adj, R^2^pred) and by the lack-of-fit test (*p* > 0.05).

Optimization was carried out using the Response Optimizer tool with the aim of maximizing oil yield. To quantify desirability, the composite desirability function (D) was used.

Comparison of two independent groups (the control sample and the optimized sample after alkaline pretreatment) was performed using parametric methods, a two-sample *t*-test or Welch’s *t*-test, depending on the results of the homogeneity-of-variance check. The normality of the distribution was assessed using the Ryan–Joiner test, and the homogeneity of variances using the F-test. Differences were considered statistically significant at *p* < 0.05.

The OriginPro 2024 software version 10.1 (OriginLab^®^, Northampton, MA, USA) was used to construct the plots.

## 3. Results and Discussion

### 3.1. Determination of Factor Levels

**Figure 1:**
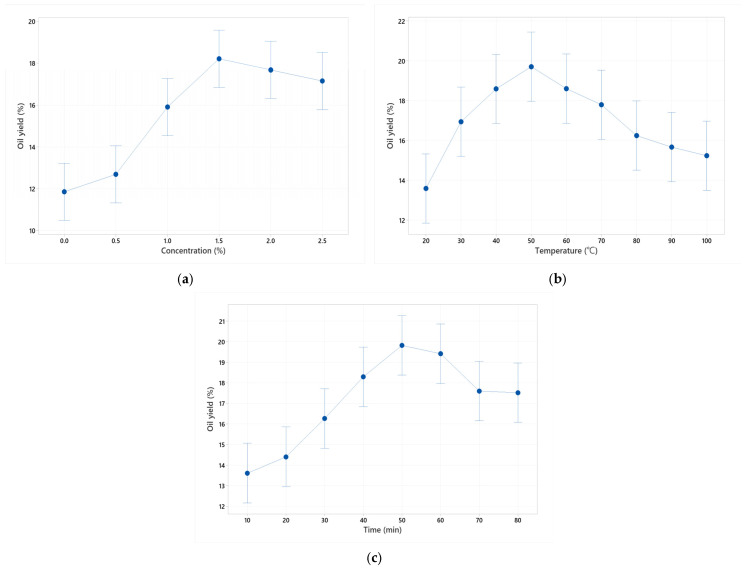
Effect of alkaline pretreatment parameters on safflower seed oil yield: (**a**) NaOH concentration, (**b**) temperature, (**c**) treatment time. Different letters within one parameter indicate significant differences (Tukey’s test, *p* < 0.05) (mean ± SD, n = 3).

To assess the effect of each technological factor on oil yield and to select working levels for subsequent process optimization, single-factor experiments were conducted to determine rational ranges for NaOH concentration, temperature, and duration of alkaline pretreatment of safflower seeds (Figure 1).

#### 3.1.1. Effect of NaOH Concentration

One-way analysis of variance revealed a significant effect of NaOH concentration on oil yield (F(5;12) = 18.41; *p* = 0.0001). The ANOVA assumptions (normality and homogeneity of variances; Bartlett’s test, *p* = 0.719) were confirmed. The post hoc Tukey test showed that the maximum oil yield, 18.21 ± 1.02%, was observed at a concentration of 1.5% NaOH. The 1.0; 1.5; 2.0; and 2.5% NaOH levels formed one homogeneous group (A) and did not differ statistically from each other (*p* > 0.05) (Figure 1a). At 0 and 0.5% NaOH, the yield was significantly lower (group B). Although the concentration of 2.5% NaOH was included in the highest homogeneous group (A), given the post hoc Tukey test, it was excluded from the final range. At this value, a slight tendency toward a decrease in the mean oil yield to 17.15 ± 1.07% was observed compared with the maximum value at 1.5%. In addition, increasing the alkali concentration above 2% led to increased reagent consumption and complication of the subsequent washing procedure. Thus, a concentration range of 1–2% NaOH was determined, which covers the levels providing oil yield values that are statistically equivalent to the maximum and is optimally centered around the efficiency peak. This range is convenient for constructing second-order designs at the stage of subsequent process optimization.

#### 3.1.2. Effect of Treatment Temperature

At a fixed NaOH concentration of 1.5% and a treatment duration of 60 min, the influence of temperature in the range of 20–100 °C was studied. The data were normally distributed, and the variances were equal (Bartlett’s test, *p* = 0.949). ANOVA confirmed a significant effect of temperature on oil yield (F(8;18) = 5.50; *p* = 0.001). The post hoc Tukey analysis demonstrated that the highest yield, 19.71 ± 1.17%, was achieved at 50 °C. The 40, 50, 60 and 70 °C levels were included in the highest homogeneous groups (A and AB) and did not differ significantly from each other, while they significantly exceeded the values at 20 °C and showed a tendency toward a decrease at temperatures above 70 °C, as reflected in Figure 1b.

As shown in the graph, the temperature factor is also an important parameter of the oil extraction process for safflower; it has been shown in [39] that thermal treatment and temperature optimization substantially affect the oil extraction percentage from safflower seeds. After reaching a maximum at 50 °C, a further increase in temperature led to a decrease in oil yield: at 60 °C, the value was 18.81%, and at 100 °C, it was 11.92%. This decrease may indicate excessive thermal exposure to the raw material, densification of the structure following treatment and drying, and partial formation of stable emulsified or bound lipid forms, as well as a possible deterioration in the pressability of the material [40], emphasizing that oil release requires overcoming two main barriers—the cell wall and the interfacial membrane of oil bodies—and that excessive application of technological factors may promote the formation of stable emulsions and hinder oil separation.

Based on these data, the range of 40–60 °C was selected for subsequent optimization as the zone of maximum efficiency without excessive thermal impact.

#### 3.1.3. Effect of Treatment Duration

After selecting the values of NaOH concentration and temperature, the influence of the alkaline treatment time from 10 to 80 min was studied. The treatment duration also significantly affected oil yield (F(7;16) = 10.65; *p* = 0.0001). The variances were equal (Bartlett’s test, *p* = 0.994), and the data followed a normal distribution. According to the Tukey test, the maximum yield, 19.82 ± 0.96%, was obtained at 50 min. The 40, 50 and 60 min levels formed the highest homogeneous groups and did not differ statistically from each other, whereas at 10–30 min and 70–80 min, the yield was significantly lower. During extended contact between the raw material and the alkali solution, the processes of leaching of soluble substances, excessive swelling, alteration of the protein–polysaccharide complex structure, and deterioration of conditions for subsequent mechanical oil recovery are intensified [14]. As a result, a portion of the oil may remain bound in the cake or pass into difficult-to-separate emulsified forms.

The range of 40–60 min was determined for further studies as providing the best yield.

Based on the results of the single-factor experiments and the post hoc Tukey analysis, it was determined that oil yield substantially depends on all three studied factors, and the following rational ranges of the independent variables were identified for subsequent optimization of the alkaline pretreatment process: NaOH concentration: 1–2%, temperature: 40–60 °C and treatment duration: 40–60 min. These ranges include the levels giving statistically maximum or near-maximum oil yield values (the highest homogeneous groups) and make it possible to avoid extreme conditions under which the yield significantly decreases. The selected ranges were used to construct the second-order Box–Behnken designs.

### 3.2. Optimization of the Conditions of Alkaline Treatment of Safflower Seeds by Response Surface Methodology (Box–Behnken Design)

#### 3.2.1. Construction and Statistical Evaluation of the Regression Model

On the basis of the single-factor screening experiments, the working ranges of the three independent variables were selected: NaOH concentration (X1: 1.0–2.0%), temperature (X2: 40–60 °C) and treatment duration (X3: 40–60 min), Table 1.

To simultaneously evaluate the linear, quadratic and interaction effects, a Box–Behnken design was used (three factors, 15 experimental points, and three central replicates) (Table 2).

The experimental oil yield values varied from 13.64 ± 0.89% to 20.65 ± 0.94% (Table 3). The CS was 11.86 ± 1.10%. The presence of a pronounced maximum in the central region of the factor space reflects the existence of a local maximum within the studied region of the factor space.

The quadratic model in uncoded units (obtained in Minitab) had the form of Equation (8):

(8) $$Y\left(\%\right)=-87.4+30.18X_{1}+1.507X_{2}+1.678X_{3}-9.995X_{1}^{2}-0.02001X_{2}^{2}\;+0.01701X_{3}^{2}-0.2{000X}_{1}X_{2}-0.16{00X}_{1}X_{3}+0.00603X_{2}X_{3}$$

where *Y* is oil yield (%), *X*_1_ is NaOH concentration (%), *X*_2_ is temperature (°C), and *X*_3_ is Time (min).

Equation (8) shows that the studied factors have a complex effect on oil yield. In particular, as the factors increase within the studied region, the positive linear coefficients increase the response values. However, the negative coefficients of the quadratic terms X_1_^2^, X_2_^2^, and X_3_^2^ indicate that the response surface has a maximum within the studied region [41].

The results of the regression analysis are presented in Table 3.

According to the data in Table 3, the most significant effects on oil yield were exerted by the NaOH concentration and treatment temperature. The linear effects of NaOH concentration and temperature were statistically significant at *p* = 0.001. The linear effect of treatment duration was *p* = 0.059, i.e., at the boundary of statistical significance. However, the quadratic effect of time was significant (*p* = 0.001), confirming the nonlinear nature of the effect of treatment duration on oil yield.

The quadratic effects of all three factors were statistically significant: for NaOH concentration, *p* = 0.000; for temperature, *p* = 0.000; and for time, *p* = 0.001. This confirms the existence of optimal factor values at which maximum oil yield is achieved. In addition, pairwise factor interactions were significant: NaOH concentration (%) × temperature (°C) (*p* = 0.007), NaOH concentration (%) × time (min) (*p* = 0.016), and temperature (°C) × time (min) (*p* = 0.043). Consequently, oil yield is determined not only by the individual action of each factor but also by their interactions.

Analysis of variance (ANOVA) (Table 4) confirmed the high statistical significance of the model (F = 39.18; *p* < 0.0001).

The coefficient of determination R^2^ was 98.60%, the adjusted R^2^ (R^2^adj) was 96.09%, and the predicted R^2^ (R^2^pred) was 96.59%. The closeness of R^2^adj and R^2^pred (difference < 0.5%) indicates the high predictive ability of the model and the absence of overfitting.

According to Table 4, the lack-of-fit was statistically insignificant (F = 0.01; *p* = 0.998). This result confirms that the model adequately describes the experimental data and that there are no systematic deviations and corresponds to the recommended criteria for assessing the quality of quadratic models in response surface methodology [37].

**Figure 2:**
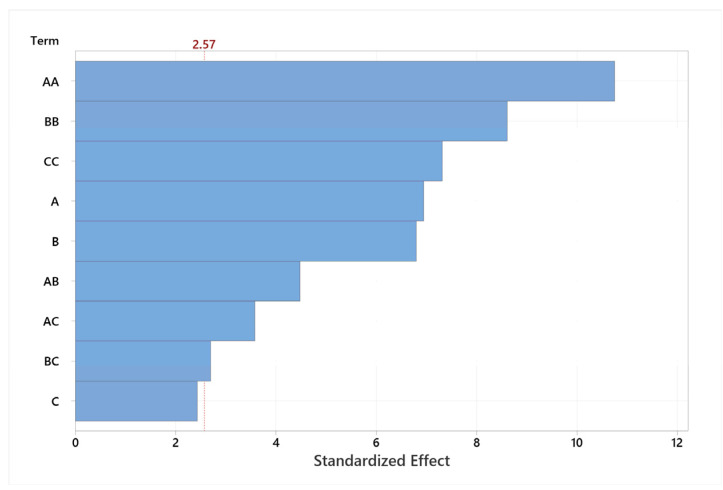
Pareto chart of the standardized effects (response is oil yield (%); α = 0.05).

The linear effects of NaOH concentration and temperature were highly significant. However, the linear effect of time (*p* = 0.059) was at the borderline of significance. Such a pattern is characteristic of quadratic models with a pronounced maximum within the factor space: strong quadratic terms create a dome-shaped form of the response surface, as a result of which the linear contribution of a factor near the optimum becomes weak and is partially masked by the curvature. At the same time, the time factor remains important owing to its highly significant quadratic effect. The model reflects this effect, which is also seen in the Pareto chart (the largest contribution comes from the quadratic terms AA, BB, and CC) (Figure 2).

Since all quadratic terms were highly significant (*p* ≤ 0.001) and had negative coefficients, it can be concluded that a clear maximum exists within the factor space [41].

All three two-factor interactions were statistically significant:

- X_1_ × X_2_ (*p* = 0.007)—Positive synergism;
- X_1_ × X_3_ (*p* = 0.016)—Negative antagonism;
- X_2_ × X_3_ (*p* = 0.043)—Weak positive synergism.

The positive X_1_ × X_2_ interaction means that increasing the temperature enhances the action of the alkali (and vice versa). This is probably related to the acceleration of the diffusion of OH^−^ ions into the seed structure, more intensive swelling of the cell-wall polysaccharides and more effective cleavage of the ester bonds of the lignin–carbohydrate complex at elevated temperatures [42].

The negative X_1_ × X_3_ interaction indicates that at high alkali concentrations, an increase in treatment duration becomes less effective. This is probably related to the risk of excessive solubilization of the cell-structure components and the formation of stable emulsions, which hinder the subsequent mechanical release of oil.

#### 3.2.2. Analysis of the Response Surfaces

**Figure 3:**
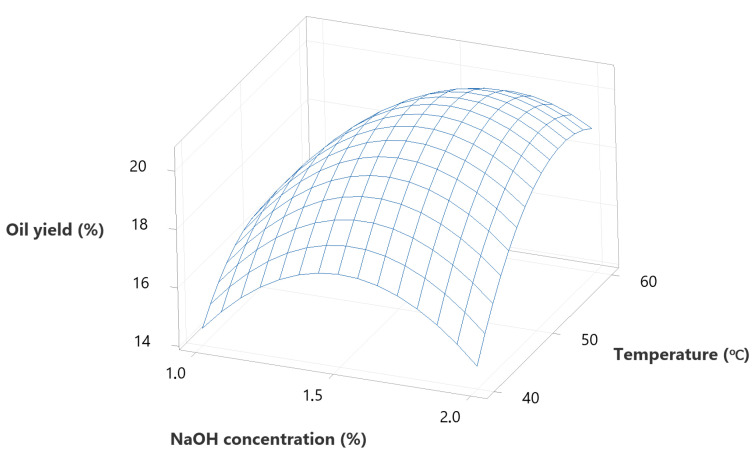
Three-dimensional response surface plot of the effects of alkali concentration (%) and treatment temperature (°C) on oil yield (%).

**Figure 4:**
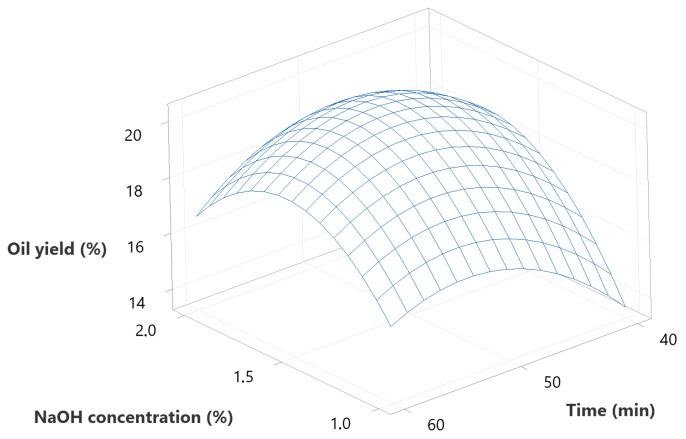
Three-dimensional response surface plot of the effects of alkali concentration (%) and treatment duration (min) on oil yield (%).

**Figure 5:**
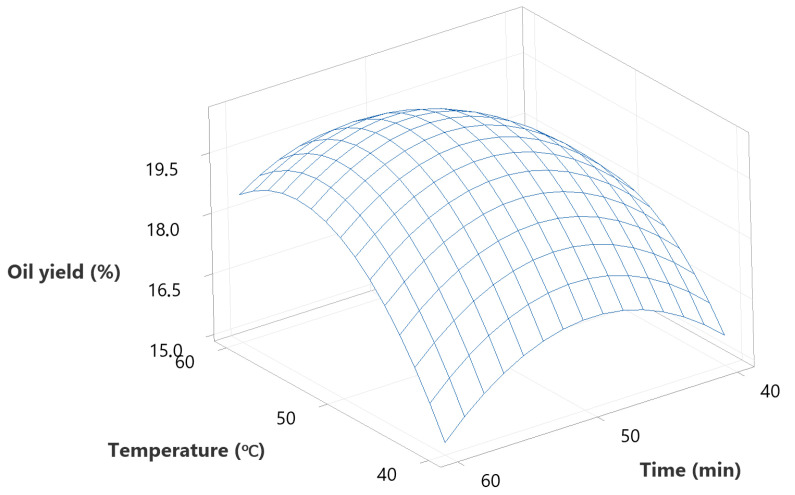
Three-dimensional response surface plot of the effects of treatment temperature (°C) and treatment duration (min) on oil yield (%).

For a more visual interpretation of the results, response surfaces (RSs) were constructed in the Minitab program, which are presented in Figure 3, Figure 4 and Figure 5. They have a dome shape with a maximum located within the studied region of the factor space and demonstrate the influence of NaOH concentration (%), temperature (°C) and time (min) on oil yield. The response surface methodology is widely used to optimize technological processes since it makes it possible to assess not only the individual influence of the factors but also their interactions, as well as to identify the nonlinear nature of the change in the response [43].

The NaOH concentration–temperature surface demonstrates the steepest rise toward the maximum, but a steep slope is observed along the NaOH concentration axis (coefficient AA = −2.499). Even a small deviation from the optimal concentration leads to a noticeable decrease in yield.

The high sensitivity to NaOH concentration is probably explained by the dual nature of the action of the alkali. At moderate values (approximately 1.0–1.7%), hydroxide ions promote swelling of the cell walls and partial destruction of the structural barriers, which facilitates the subsequent mechanical release of oil. When the optimal concentration is exceeded, the risk of denaturation of the membrane proteins oleosins and thinning of the interfacial membrane increases [44], which can lead to the formation of stable emulsions and hinder oil separation. A similar “turning-point” dependence on alkali concentration is characteristic of the alkaline pretreatment of lignocellulosic biomass, where an excess of reagent leads to enhanced solubilization of components and loss of mass of the target material [42].

The surfaces involving time are “gentler”, which is consistent with the lower significance of the linear effect of time.

The shape of the surfaces shows that the optimum is located within the studied region. This creates the prerequisites for possible scaling-up of the process within the studied ranges of parameters.

#### 3.2.3. Optimization and Validation of the Optimization Results

**Figure 6:**
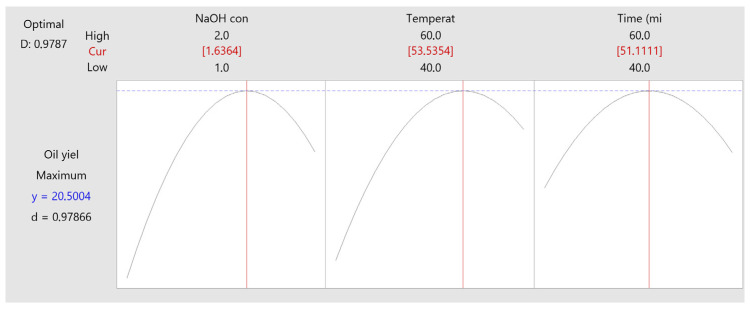
Response Optimizer plot indicating the optimal pretreatment conditions for maximum oil yield.

Using the desirability function in the Response Optimizer tool of the Minitab statistical data-processing software package (objective—maximization of oil yield), the optimal conditions of alkaline pretreatment of safflower seeds were obtained: NaOH concentration = 1.64%, temperature = 53.54 °C, and time = 51 min. Predicted yield = 20.50% (95% CI: 19.87–21.13%), and composite desirability D = 0.9787 (Figure 6).

The obtained value of the desirability function is close to unity, which indicates a high degree of correspondence to the set optimization objective.

The confirmation experiment was carried out at the following parameters: NaOH concentration = 1.64%, temperature = 53.5 °C, time = 51 min. This was due to the technical characteristics of the laboratory equipment used for validation. The validation tests were carried out in triplicate. This experiment showed an actual OS yield of 21.47 ± 0.17%, which is in good agreement with the prediction (deviation < 5%) and lies within the 95% prediction interval. Compared with the CS of 11.86 ± 1.10%, the increase was 9.61 absolute percent. The oil extraction efficiency increased by 37.52%, from 46.31% to 83.83%.

The obtained results suggest that under optimal conditions, alkaline pretreatment promotes moderate swelling and partial destruction of the structural barriers of the seed coat, as well as facilitating the release of oil. Exceeding the optimal values of the parameters probably leads to excessive solubilization of the components and the formation of stable emulsions, which reduces the efficiency of mechanical pressing. The obtained results are consistent with the data on the thermal pretreatment of safflower seeds using RSM [39].

Thus, response surface methodology (Box–Behnken) in combination with alkaline pretreatment made it possible not only to determine the optimal conditions but also to quantitatively describe the complex nonlinear and interaction effects, which creates the prerequisites for using the model for possible scaling-up of the process.

### 3.3. Determination of the Main Physicochemical and Structural Characteristics of Safflower Seeds and Oil Before and After Pretreatment

The main physicochemical characteristics of the seeds before and after alkaline pretreatment are presented in Table 5.

As can be seen from Table 5, a statistical comparison of the residual alkali indicator is not possible since this indicator is absent in the control sample. In OS, the residual alkali content was 0.665 ± 0.02 mg/g. The obtained data indicate that after treatment and subsequent washing, a certain amount of alkali is retained in the seeds. This is associated with the fact that during contact of plant raw material with the alkali solution, the alkali not only acts on the surface but also partially penetrates the outer layers of the hull. As a result, alkali may be retained in the porous structure of the seeds, particularly in hull regions where tissue loosening and alteration of the surface component structure occur. In the literature [18,19], it is noted that NaOH is capable of dissolving surface waxes, acting on cell walls, partially removing hemicelluloses and lignin, and increasing the porosity and accessibility of the plant material structure. These changes may contribute to better disruption of the hull and facilitate oil release during subsequent cold pressing. However, at the same time, the need for control of residual alkali becomes more important, as its excess may adversely affect the quality of the treated seeds and the oil obtained.

It should be noted that the detected residual alkali content does not imply inefficiency of the washing procedure but rather confirms the very fact of NaOH interaction with the surface layers of safflower seeds. In this context, the value of 0.665 mg/g can be considered an important technological control parameter. It allows for an assessment of how completely the alkali is removed after treatment and, if necessary, adjustment of the washing protocol since this indicator is directly related to the quality and safety of the treated raw material.

A comparison of the hull content of safflower seeds before and after alkaline pretreatment under optimal conditions revealed statistically significant differences (two-sample *t*-test, *p* = 0.012). In CS, the hull content was 49.22 ± 0.38%, whereas after alkaline treatment in OS, this indicator decreased to 47.35 ± 0.63%. The difference was 1.87 absolute percent, which indicates a noticeable decrease in the proportion of the hull after treatment. Such a decrease is presumably explained by the action of the alkali solution on the surface tissues of the seeds and the partial removal of waxy, pectin and other surface components of the hull; delignification; and the weakening of the cell structure of the hull. As a result, the hull in OS became less dense and more accessible to mechanical action. This result is of practical significance, as oil in safflower seeds is predominantly concentrated in the kernel, while the hull can impede its release. Therefore, the reduction in hull content after treatment can be regarded as one of the factors contributing to improved cold pressing efficiency.

### 3.4. Assessment of the Seed Hull by Fourier-Transform Infrared Spectroscopy (FTIR)

**Figure 7:**
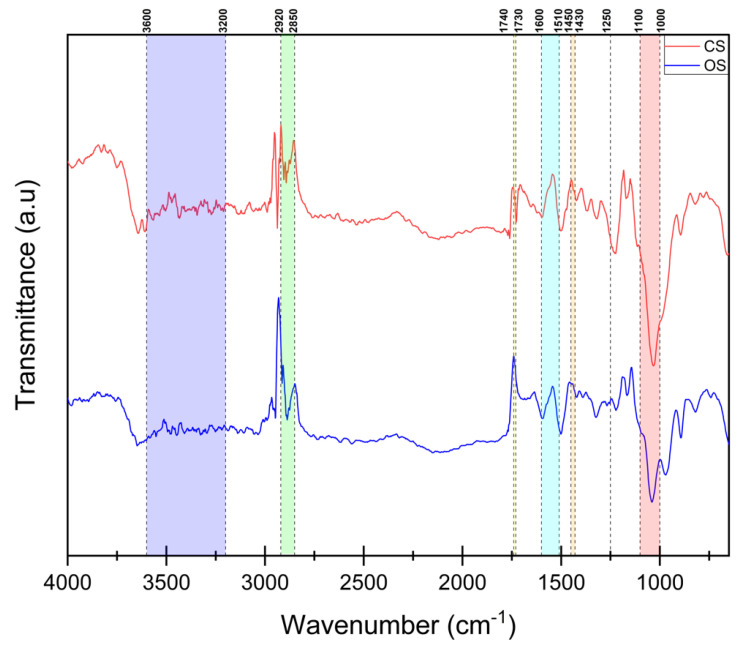
FTIR spectra of the safflower seed hull CS and OS.

Figure 7 presents the FTIR spectra of safflower seed hulls before and after optimized alkaline treatment. Overall, both spectra display a similar pattern, indicating preservation of the basic lignocellulosic nature of the seed hull. At the same time, after alkaline treatment, the intensities of individual absorption bands change noticeably, particularly in regions associated with hydroxyl, aliphatic, carbonyl, and ester groups.

In the region of 3600–3200 cm^−1^, a broad band characteristic of O-H stretching vibrations is observed. These groups may belong to cellulose, hemicellulose, residual moisture, and phenolic fragments of lignin. According to literature data, the broad band in this region is typically associated with inter- and intramolecular hydrogen bonding in plant raw materials and cell wall components [45]. On the OS spectrum, the shape of this band changes somewhat, which may be associated with partial disruption of hydrogen bonds and loosening of the hull structure under the action of the alkali.

In the region of 2920–2850 cm^−1^, pronounced bands of C-H stretching vibrations in methyl and methylene groups are observed. Such bands are characteristic of aliphatic fragments of waxes, lipids, suberin, and lateral chains of lignin. In the literature, peaks around 2920 and 2850 cm^−1^ are attributed to asymmetric and symmetric vibrations of C-H groups in aliphatic chains [45]. On the OS spectrum, this region appears more defined, which may indicate not an increase in lipid content but rather an alteration in the accessibility of these groups following removal of some surface impurities, waxes, and loosely bound hull components.

The band in the region of approximately 1730–1740 cm^−1^ is typically assigned to C=O vibrations of ester, carboxyl, and acetyl groups of hemicellulose, as well as fragments of lignin and the plant cuticle. In FTIR studies of plant materials, the peak near 1735 cm^−1^ is associated with carbonyl, acetyl, and uronic acid ester groups of hemicellulose [46]. The change in this region on the OS spectrum can be explained by partial saponification of ester bonds and removal of some hemicellulosic or waxy components. This is in good agreement with the mechanism of alkali action on plant raw materials: alkaline treatment is capable of weakening the bonds between lignin, hemicellulose, and cellulose and of removing some extractive substances [45,47].

In the range of 1600–1510 cm^−1^, bands associated predominantly with the aromatic skeleton of lignin are observed. Changes in this region on the OS spectrum indicate modification of the lignin component in the hull. For plant biomass, the band around 1510–1512 cm^−1^ is often used as a diagnostic signal for lignin, while the region around 1600–1630 cm^−1^ may reflect aromatic vibrations of lignin [47].

Changes in the region around 1430 cm^−1^ can be associated with C-H deformation vibrations of cellulose. On the OS spectrum, this band is more pronounced compared with CS, which is probably related to the loosening of the hull and partial removal of associated components such as hemicellulose, pectic substances, and lignin, as a result of which the cellulose fraction becomes more discernible in the spectra. Such changes in the 1430–1450 cm^−1^ region are generally considered indicative of structural changes in the cellulose fraction of plant raw materials [46]. Bands around 1250 cm^−1^ are typically associated with disruption of C-O bonds characteristic of lignin and hemicellulose; on the CS spectrum, this band is more pronounced, while its intensity changes on the OS spectrum. Analogous changes have been described in [20] for hazelnut shells, where the weakening of the band around 1250 cm^−1^ was attributed to alterations in lignin and hemicellulose, while the more pronounced region around 1030 cm^−1^ was associated with the polysaccharide fraction of the raw material. The most noticeable changes on the OS spectrum are observed in the region of 1100–1000 cm^−1^, where C-O, C-O-C, and glycosidic bond vibrations of cellulose and hemicellulose are manifested. This region becomes more pronounced, which may be associated with loosening of the hull and alteration of its polysaccharide structure.

Thus, FTIR analysis showed that optimized alkaline treatment leads to noticeable changes in the structure of the safflower seed hull. Weakening of the bands associated with hemicellulose, pectic substances, and the lignin component indicates partial disruption of the lignocellulosic complex. At the same time, more pronounced bands in the polysaccharide region indicate that the cellulose and hemicellulose fractions of the hull become more accessible in the spectra. Such changes can be associated with loosening of the hull and a reduction in its strength. This explains the increase in oil yield during cold pressing: after pretreatment, the hull is more readily disrupted, and oil is more efficiently released from the seeds.

### 3.5. Oil Quality

The physicochemical, color and antioxidant characteristics of CS oil and OS oil are presented in Table 6. All determinations were performed in triplicate. Alkaline pretreatment had a complex effect on oil quality.

All determinations were carried out in triplicate, and the mean value ± standard deviation (SD) is reported; n = 3.

As can be seen from Table 6, compared with CS oil, the oil yield of OS oil increased by 9.61 percentage points relative to the initial value of 11.86 ± 1.10%. At the same time, the degree of oil extraction increased from 46.31 to 83.83%, that is, by 37.52 percentage points, which confirms more efficient oil release from the pretreated seeds.

The color characteristics of CS oil and OS oil are presented in Table 6. The color of the studied samples was, for CS oil, L* = 34.43, a* = −0.66, and b* = 30.09, and for OS oil, L* = 35.28, a* = −0.13 and b* = 31.16. The high positive b* values indicated a pronounced predominance of the yellow component, whereas the near-zero negative a* values indicated a slight shift of the hue toward the green region. In general, statistically significant increases were observed (*p* = 0.021; *p* < 0.001 and *p* = 0.005). The obtained values are consistent with literature data for safflower oil [27]; it is also known that the color intensity depends on the safflower cultivar, the content of natural pigments and the oil extraction methods.

As can be seen from Table 6, the acid value in OS oil decreased more than 2.5-fold, from 1.29 ± 0.14 to 0.48 ± 0.16 mg KOH/g (*p* = 0.003) compared with CS oil, which is a favorable change. Such a decrease is probably related to the partial removal of free fatty acids during the alkaline treatment of the seeds. It is known that with a decrease in the acid value, free acids decrease, improving the quality of the obtained product and reducing the severity of hydrolytic changes in the lipids, which is consistent with earlier studies by Aydeniz et al. (2014) [27].

As can be seen from Table 6, the peroxide value also significantly decreased, from 1.42 ± 0.02 to 1.03 ± 0.04 meq O_2_/kg, *p* < 0.001. A lower content of primary oxidation products indicates better oxidative stability of the freshly pressed oil [48]. Possible reasons include the faster and more complete release of oil during pressing and the removal of part of the prooxidant compounds during washing. This indicates that the alkaline treatment of the seeds under the selected conditions did not cause a pronounced accumulation of primary oxidation products. The obtained values are comparable with literature data for safflower oil; however, this indicator depends on the cultivar, the extraction method and the storage conditions [49].

In Table 6, a small but statistically significant decrease in the saponification value was observed, from 191.24 ± 0.08 to 189.84 ± 0.21 mg KOH/g, *p* < 0.001. This indicates the absence of a pronounced change in the overall composition of the saponifiable lipid fraction. At the same time, the saponification value does not allow for a direct assessment of soap formation; therefore, the obtained results do not exclude partial hydrolysis or neutralization of free fatty acids during treatment [50].

The total content of phenolic compounds (TPC) decreased from 44.30 ± 0.92 to 36.31 ± 1.05 mg GAE/kg (*p* = 0.001). Accordingly, the antioxidant activity of DPPH and ABTS also decreased. The decrease in total phenolic compounds is probably related to the alkaline pretreatment of the safflower seeds. According to earlier studies, pretreatment of safflower seeds changes the content and composition of phenolic compounds in the resulting oil [27]; the total content of phenolic compounds in safflower oil can also change depending on the safflower seed cultivar, which is confirmed by the differences in phenolic compounds between oils obtained from different safflower cultivars [8]. In addition, the phenolic content can also be influenced by such factors as the oil extraction methods, climatic conditions and oil storage conditions [32].

The main fatty acid in the studied samples of safflower oil (Table 6) was linoleic acid, the content of which was 78.27% in CS oil and 78.35% in OS oil. The proportion of oleic acid decreased slightly, from 10.89 ± 0.01 to 10.39 ± 0.71%, whereas the content of palmitic and stearic acids increased, respectively, from 6.96 ± 0.23 to 7.21 ± 0.42% and from 2.67 ± 0.01 to 2.90 ± 0.28%. The total content of polyunsaturated fatty acids practically did not change and amounted to 78.35–78.40%. No statistically significant differences were found for the main fatty acids (C16:0, C18:0, C18:1, C18:2, etc.). The obtained results indicate that pretreatment of safflower seeds with an aqueous NaOH solution did not have a pronounced effect on the fatty acid composition of the oil. Both samples were characterized by a high content of linoleic acid and belonged to the high-linoleic type of safflower oil. In [27], after pretreatment of safflower seeds, no significant differences in fatty acid composition were observed in oils obtained by cold pressing.

The obtained oil corresponds to the ranges of the main fatty acids provided for by the Codex Alimentarius Commission (FAO/WHO) [51].

## 4. Conclusions

The conducted study demonstrated that the dense lignified seed coat of safflower is one of the key factors limiting oil yield during cold pressing. Controlled alkaline pretreatment makes it possible to partially modify the structure of the outer seed tissues, weaken the barrier properties of the coat, and increase the accessibility of the lipid fraction for mechanical extraction.

Based on the results of the single-factor experiments (Tukey’s post hoc analysis), the influence of all three studied factors on oil yield was established. This made it possible to determine rational ranges of the independent variables for optimizing the alkaline pretreatment process: NaOH concentration of 1–2%, temperature of 40–60 °C, and treatment duration of 40–60 min.

Using the response surface methodology and a Box–Behnken design, a statistically significant quadratic model was obtained (R^2^ = 98.60%, non-significant lack-of-fit), which adequately describes the effect of the studied technological parameters on oil yield. The optimal pretreatment conditions were as follows: NaOH concentration of 1.64%, temperature of 53.54 °C, and duration of 51.11 min. Under these parameters, the actual oil yield reached 21.47 ± 0.17% (model prediction—20.50%), and the oil extraction coefficient increased by 37.52 percentage points (from 46.31% to 83.83%). The obtained experimental result lies within the 95% prediction interval of the model.

The alkaline treatment led to a statistically significant reduction in seed coat content (from 49.22 ± 0.38% to 47.35 ± 0.63%, *p* = 0.012) and a residual alkali content of 0.665 ± 0.02 mg/g after washing. FTIR spectroscopy confirmed the partial destruction of the lignocellulosic complex of the coat (attenuation of the bands associated with hemicellulose, lignin, and ester groups), which is consistent with the increased efficiency of cold pressing.

An important result is the improvement of a number of oil quality indicators: the acid value decreased more than 2.5-fold (from 1.29 ± 0.14 to 0.48 ± 0.16 mg KOH/g, *p* = 0.003), and the peroxide value decreased from 1.42 ± 0.02 to 1.03 ± 0.04 meq O_2_/kg (*p* < 0.001). The fatty acid composition remained practically unchanged, with a predominance of linoleic acid (78.27%). At the same time, a moderate decrease in the content of phenolic compounds and in antioxidant activity was observed.

Thus, the proposed alkaline pretreatment regime provides a substantial increase in oil yield while preserving and even improving the key indicators of its quality.

The present study made it possible to establish the optimal conditions for the alkaline pretreatment of safflower seeds and to quantitatively describe the nonlinear and interactive effects of NaOH concentration, temperature, and treatment duration on oil yield. At the same time, a number of limitations related to the scale and scope of the experimental work should be taken into account when interpreting the results and planning further research.

The optimization was carried out with respect to a single criterion—oil yield. Although additional analyses (fatty acid profile, phenolic compound content, antioxidant activity, color, acid value, and peroxide value) showed the absence of any negative effect of the optimal regime on oil quality, a full multi-criteria optimization that simultaneously accounts for oxidative stability and other quality indicators remains a task for subsequent work. The model was constructed at fixed values of the initial seed moisture content and the pressing parameters, which limits the scope of its direct generalization.

The experiments were performed on a laboratory screw press of a single type and on a single safflower cultivar (Moldir-2008). Therefore, the applicability of the identified optimal conditions to presses of different geometry, to other pressure regimes, and to cultivars with different coat thickness or oil content requires separate verification. Neither a quantitative separation of the contributions of the physical loosening of the coat and chemical changes in the cell walls nor a complete material balance (including the loss of soluble organic substances during washing) was carried out within the framework of this study.

Future research should be directed toward the following areas. First, conducting a multi-criteria optimization that includes oil quality indicators and oxidative stability. Second, pilot-scale validation of the optimal regime on equipment of various configurations, with simultaneous assessment of energy consumption, material balance, and methods for the disposal of alkaline wastewater. Third, extending the study to other safflower cultivars and a deeper investigation of the mechanisms of alkali action using electron microscopy, quantitative FTIR analysis, and modern methods for assessing the state of proteins and lipids. Implementation of these stages will make it possible to move from laboratory optimization to a technologically and environmentally sound process.

The results obtained in the present work provide a solid foundation and valuable evidence to support and facilitate such a transition.

## Figures and Tables

**Table 1:** Values of the three factors at three coding levels.

Levels	Variables		
	X_1_ (NaOH Concentration, %)	X_2_ (Temperature, °C)	X_3_ Time (Min)
−1	1.0	40	40
0	1.5	50	50
1	2	60	60

**Table 2:** Box–Behnken experimental design showing the values of the variables and the experimental data on oil yield (mean ± SD, n = 3).

Trial No.	NaOH Concentration (%)	Temperature (°C)	Time (Min)	Oil Percentage (%)
Control	-	-	-	11.86 ± 1.10
1	1.5	50	50	20.43 ± 1.24
2	1.5	50	50	20.65 ± 0.94
3	2	50	40	17.51 ± 1.02
4	1.5	60	40	16.49 ± 0.80
5	2	50	60	16.64 ± 0.95
6	1.5	40	40	15.59 ± 1.12
7	1	50	40	13.64 ± 0.89
8	1	40	50	14.48 ± 0.95
9	1.5	60	60	18.49 ± 1.31
10	1	50	60	15.98 ± 0.91
11	2	40	50	14.61 ± 1.33
12	1.5	40	60	15.18 ± 1.18
13	2	60	50	18.81 ± 0.58
14	1	60	50	14.67 ± 1.19
15	1.5	50	50	19.34 ± 0.78

All independent measurements were performed in triplicate; data are presented as mean ± standard deviation (SD).

**Table 3:** Response surface regression: oil yield (%) versus NaOH concentration (%); temperature (°C); time (min).

Term	Coefficient	SE Coefficient	T-Value	*p*-Value	VIF
Constant	20.140	0.258	78.09	0.000	
NaOH concentration (%)	1.098	0.158	6.95	0.001	1.00
Temperature (°C)	1.074	0.158	6.80	0.001	1.00
Time (min)Time (min)	0.384	0.158	2.43	0.059	1.00
NaOH concentration (%) × NaOH concentration (%)	−2.499	0.232	−10.75	0.000	1.01
Temperature (°C) × Temperature (°C)	−2.001	0.232	−8.61	0.000	1.01
Time (min) × Time (min)	−1.701	0.232	−7.32	0.001	1.01
NaOH concentration (%) × Temperature (°C)	1.000	0.223	4.48	0.007	1.00
NaOH concentration (%) × Time (min)	−0.800	0.223	−3.58	0.016	1.00
Temperature (°C) × Time (min)	0.603	0.223	2.70	0.043	1.00

**Table 4:** Analysis of variance for the oil yield regression model.

Source	Degree of Freedom	Adjusted Sum of Squares	Adjusted Mean Square	F Value	*p* Value
Model	9	70.3669	7.8185	39.18	0.000
Linear	3	20.0377	6.6792	33.47	0.001
NaOH concentration (%)	1	9.6361	9.6361	48.29	0.001
Temperature (°C)	1	9.2235	9.2235	46.22	0.001
Time (min)	1	1.1781	1.1781	5.90	0.059
Square	3	42.3172	14.1057	70.69	0.000
NaOH concentration (%) × NaOH concentration (%)	1	23.0539	23.0539	115.53	0.000
Temperature (°C) × temperature (°C)	1	14.7877	14.7877	74.10	0.000
Time (min) × time (min)	1	10.6865	10.6865	53.55	0.001
2-way interaction	3	8.0120	2.6707	13.38	0.008
NaOH concentration (%) × temperature (°C)	1	4.0000	4.0000	20.04	0.007
NaOH concentration (%) × time (min)	1	2.5600	2.5600	12.83	0.016
Temperature (°C) × time (min)	1	1.4520	1.4520	7.28	0.043
Error	5	0.9978	0.1996		
Lack-of-fit	3	0.0136	0.0045	0.01	0.998
Pure error	2	0.9842	0.4921		
Total	14	71.3647			

**Table 5:** Main physicochemical characteristics of safflower seeds before and after alkaline pretreatment.

Parameter	CS	OS
Residual alkali, mg/g	-	0.665 ± 0.02
Hull content, %	49.22 ± 0.38	47.35 ± 0.63
Acid value, mg KOH/g	1.29 ± 0.14	0.48 ± 0.16

All determinations were carried out in triplicate, and the mean value ± standard deviation (SD) is reported.

**Table 6:** Physicochemical indicators of CS oil and OS oil.

Indicators	CS Oil	OS Oil
Oil yield, %	11.86 ± 1.10	21.47 ± 0.17
Color		
L*	34.43 ± 0.17	35.28 ± 0.28
a*	−0.66 ± 0.05	−0.13 ± 0.03
b*	30.09 ± 0.09	31.16 ± 0.26
Acid value, mg KOH/g	1.29 ± 0.14	0.48 ± 0.16
Peroxide value, O_2_/kg	1.42 ± 0.02	1.03 ± 0.04
Saponification value, mg KOH/g	191.24 ± 0.08	189.84 ± 0.21
TPC, mg GAE/kg	44.30 ± 0.92	36.31 ± 1.05
DPPH (µm Trolox/kg)	52.56 ± 1.05	47.83 ± 0.93
ABTS (µm Trolox/kg)	81.72 ± 0.86	65.38 ± 0.54
**Fatty acid, %:**		
C14:0 (Myristic acid)	0.11 ± 0.05	0.12 ± 0.08
C16:0 (Palmitic acid)	6.96 ± 0.23	7.21 ± 0.42
C18:0 (Stearic acid)	2.67 ± 0.01	2.90 ± 0.28
C18:1 (Oleic acid)	10.89 ± 0.01	10.39 ± 0.71
C18:2 (Linoleic acid)	78.27 ± 0.37	78.35 ± 1.69
C18:3 (Alpha-linolenic acid)	0.08 ± 0.03	0.05 ± 0.02
C20:1 (Eicosenoic acid)	0.17 ± 0.06	0.19 ± 0.09
SFA	9.74	10.23
MUFA	11.06	10.58
PUFA	78.35	78.4

## Data Availability

The original contributions presented in this study are included in the article. Further inquiries can be directed to the corresponding author.

## Abbreviations

The following abbreviations are used in this manuscript:

CS	Control Sample
OS	Optimized Sample
FTIR	Fourier-Transform Infrared Spectroscopy
RSM	Response Surface Methodology
ANOVA	Analysis of Variance
